# On the Possible Chemical Justification of the Ethnobotanical Use of *Hyptis obtusiflora* in Amazonian Ecuador

**DOI:** 10.3390/plants7040104

**Published:** 2018-11-23

**Authors:** Carmen X. Luzuriaga-Quichimbo, José Blanco-Salas, Carlos E. Cerón-Martínez, Milan S. Stanković, Trinidad Ruiz-Téllez

**Affiliations:** 1 Centro de Investigación Biomédica, Facultad de Ciencias de la Salud Eugenio Espejo, Universidad Tecnológica Equinoccial, Av. Mariscal Sucre y Mariana de Jesús, Quito 170527, Ecuador; luzuriaga.cx@gmail.com; 2 Department of Vegetal Biology, Ecology and Earth Science, Faculty of Sciences, University of Extremadura, 06071 Badajoz, Spain; truiz@unex.es; 3 Herbario Alfredo Paredes, QAP, Universidad Central de Ecuador, Quito 170129, Ecuador; cecm57@yahoo.com; 4 Department of Biology and Ecology, Faculty of Science, University of Kragujevac, 34000 Kragujevac, Serbia; mstankovic@kg.ac.rs

**Keywords:** *Hyptis*, Lamiaceae, Kichwa, terpene, caryophyllene, xanthine oxidase, gut, anti-inflammatory, uricosuric

## Abstract

In rural areas of Latin America, *Hyptis* infusions are very popular. *Hyptis obtusiflora* extends from Mexico throughout Central America to Bolivia and Peru. It has added value in Ecuador where it has been used by different ethnic groups. We aimed to learn about the traditional knowledge of ancient Kichwa cultures about this plant, and to contrast this knowledge with the published information organized in occidental databases. We proposed to use traditional knowledge as a source of innovation for social development. Our specific objectives were to catalogue the uses of *H. obtusiflora* in the community, to prospect on the bibliography on a possible chemical justification for its medicinal use, to propose new products for development, and to give arguments for biodiversity conservation. An ethnobotanical survey was made and a Prisma 2009 Flow Diagram was then followed for scientific validation. We rescued data that are novel contributions for the ethnobotany at the national level. The catalogued main activity of anti-inflammation can be related to the terpene composition and the inhibition of xanthine oxidase. This opens the possibility of researching the extract of this plant as an alternative to allopurinol or uricosuric drugs. This is a concrete example of an argument for biodiversity conservation.

## 1. Introduction

The genus *Hyptis* is the second most important of the American Lamiaceae. It is made up of at least 290 species which are almost exclusively Neotropical [1,2]. Plants of this genus are covered with glandular trichomes that produce essential oils that make them very popular in rural areas of Latin America where they are taken as an infusion to treat respiratory and gastrointestinal disorders or skin diseases [3,4]. Different pharmacological activities (antibacterial [5,6] anti-inflammatory [7], antiparasitic [8], and antiproliferative [9]) have been tested experimentally in this genus, so it is an interesting taxon in the field of medical applications. Some *Hyptis* have been used against malaria (*Hyptis mutabilis* (Rich.) Briq. [10]), and others have been used against scabies (*Hyptis suaveolens* (L.) Poit. [11]) or as insect repellents (*Hyptis tafallae* Benth [12]). Nearly 20 species have been reviewed from the phytochemical point of view [13]. The volatile oil is rich in monoterpenes and sesquiterpenes and have different compositions characterized by the occurrence of major components such as E-caryophyllene, 1,8-cineole, and sabinene or others such as eugenol or cadinene. Minor constituents, which are almost always present, are *p*-cymene and α-pinene [13].

*Hyptis* is the best assorted genus of the genuine, useful Ecuadorian Lamiaceae. In Ecuador, the majority of Lamiaceae plants with catalogued uses are allochthonous. Contrary to this, there are 10 autochthonous *Hyptis* species included in the list of useful plants from Ecuador [14]. 

They have interesting chemical compounds and biological activities as reported by experimental works published in the scientific literature (Table 1), but their traditional knowledge is mostly reduced to small ethnic groups and areas, and are either endangered or near extinct [14].

**Figure 1:**
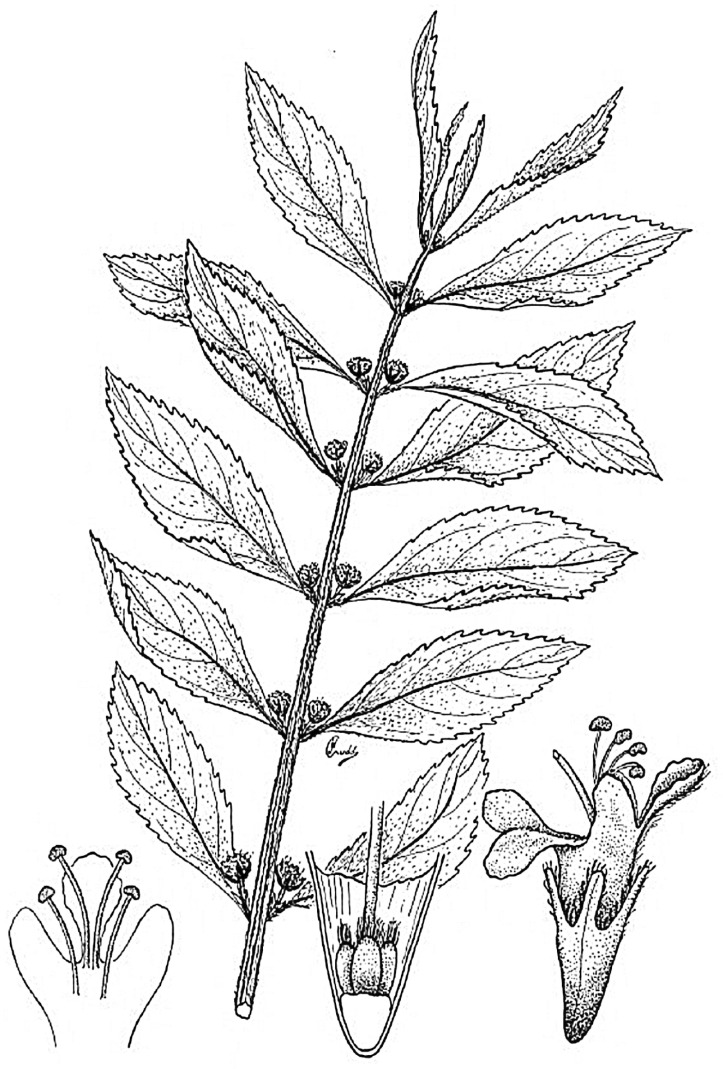
*Hyptis obtusiflora* [28].

The species with a more widespread use in the Ecuadorian catalogue is *Hyptis obtusiflora* (Figure 1), perhaps because of a certain colonizing character and a facility to occupy habitats of tropical rain forests. In Ecuador, it has been collected from several locations of the Coast and Sierra Regions and in the northern-most Amazonian provinces such as Orellana or Napo. It can be found from sea level to the montane (0–1800 m). Its natural distribution area extends north from Peru (where it was first described [25]) throughout Central America to Mexico [26]. In Peru, the national inventory of the Ministry on Traditional Medicine picks up its use against ringworms, head wounds, and pharyngitis [27], which comes from the Amazonic Yanesha populations who have an ancestral knowledge of the plant they called “ollamepan pasheñorrer”. There is very little registered bibliography from other countries.

In the frame of the Convention on Biological Diversity and the corresponding procedures of the Nagoya Protocol [29,30], we have considered engaging in the contribution of arguments for biodiversity conservation in Amazonian Ecuador in order to deepen the knowledge of and research on *Hyptis obtusiflora*. We have selected the scarcely contacted habitats of River Bobonaza to learn about the traditional knowledge of ancient Kichwa cultures about this plant and to justify the applications of the species through a critical bibliographic prospection from the experimental sciences perspective. We propose to use traditional knowledge as a source of innovation for social development.

For all these reasons, we proposed, as specific objectives of this paper:

1. To catalogue some traditional and threatened uses of *H. obtusiflora*,
2. To justify the reasons for its consumption by bibliographic prospection,
3. To propose new research or products for development, and
4. To give arguments for biodiversity conservation.

## 2. Results and Discussion

- *On the Singularity of the Studied Plant and the Value of the Surveyed Ethnobotanical Community*

The conservation of traditional knowledge allows us to report on real testimonies of little-contacted populations located in the Amazon, whose life has developed around plant biodiversity and the use of floristic resources. However, this work fulfills one of the goals of the Ecuador National Biodiversity Research Agenda dealing with prioritizing research in those gaps of knowledge on Ecuador’s biodiversity [31].

*Hyptis obtusiflora* (“mule shell” or “secret of Indian” in Spanish, “taku taku” in Awapit or “waka muké” in Shuar-Chicham) had not been previously collected nor cited in the Bobonaza Basin, which occupies most of Pastaza, the biggest province of the country (29.641 km^2^); this paper is a first advance. Regardless of this, in Ecuador, the Mestizo population uses its juice to heal wounds, and in Pichincha province, infusions are made for hot baths; the Awa from Carchi bath their legs with the ashes made from the burning the plant; the Shuar of Napo and Orellana cook the leaves to relieve the flu and to fight skin infections; and the Chachis of Esmeraldas prepare drinks with macerated leaves to relieve stomach pain [14].

Our field work revealed that the Kichwa of the Bobonaza in Pakayaku used *Hyptis obtusiflora* externally as a medicinal plant on the skin to treat stings, pimples, or injuries that insects cause, especially in the most vulnerable individuals of the population, i.e., the children. They use the juice of the leaf, and the guideline is a twice-a-day application. The people of the community value this remedy very positively. The transmission of this knowledge has occurred orally in the ayllus (families) through specific conversations among women. It is a current use and very often used at the frequency with which these injuries occur in the location where the climate promotes an abundance of insects. The plant is consumed just after collection; it has never been stored or preserved.

The data we collected had not been documented among the Kichwas ethnic group in the country. These aspects and the vernacular name given in the community (karacha panga) in the Kichwa language are novel contributions for ethnobotany at the national level. The conservation and sustainable use of biological diversity, particularly of little-known species in vulnerable ecosystems such as the tropical forests of these communities of Pastaza, which remains nowadays underexplored from the botanical point of view [32], is a worthwhile task.

- Towards an Explanation of the Use of the Plant: Xanthine Oxidase (XO) Inhibition

Xanthine oxidase (XO) activates inflammatory processes [33] by stimulating the production of reactive oxygen species [34]. The metanolic extract (1 µg/mL) of this plant has been demonstrated to inhibit 40% of XO enzymatic activities [35]. The extract of *H. obtusiflora* can be considered anti-inflammatory for this reason.

The specific chemical profile of *Hyptis obtusiflora* has not yet been studied, although there is information about co-generic species and data on their chemical profiles (see Table 1). The biological applications of some of the components isolated from close species are well known as anti-inflammatories or antinociceptives in several cases. Thus, 1,8-cineole has shown therapeutic benefits in inflammatory diseases, such as asthma and chronic obstructive pulmonary disease [36]; E-caryophyllene is an antiinflammatory molecule because (a) it produces an anti-spasmodic activity on smooth muscle, which is probably related to Ca^2+^ channel blockade [37]; and (b) it induces inhibition of cytokines involved in the arachidonic acid and histamine pathways [38].

Finally, we must emphasize how the inhibitory activity of XO that was firstly tested at biochemical laboratories [35] has recently been tested in silico using docking software [34]. This virtual approach has revealed that the inhibitory enzymatic action is related to the structural affinity of XO and E-caryophyllene [34]. This is consistent with the foregoing [38] experimental publications.

- Innovative and Clinical Possibilities for H. obtusiflora Use and Conservation under the Nagoya Protocol

Innovative drug discovery in anti-inflammatories today is focused in non-purine structures which offer some advantages over existing commercialized molecules such as allopurinol. To date, the alternative drug had been febuxostat, but the USA (Food and Drug Administration) FDA recently issued safety alerts for its use [39]. This research speciality is oriented to treat hyperuricemia, gout, ulcers, cancer, ischemia, hypertension, and oxidative damage [40]. For this reason, it seems very interesting to carry out new research lines focused on the characterization of the chemical profile of the essential oil and the chemotypes of *Hyptis obtusiflora* in the Kichwa communities of the Bobonaza River Basin in particular and the Amazonian Ecuador in general. It is a very useful plant to be cultivated because it is an autochthonous element with an easy method of propagation, and the essential oil can give many medicinal benefits. The pharmacological activity of the components dealing with XO inhibition and anti-inflammatory processes must be quantified and standardized. This is a good case for demonstrating the possibilities that vegetal biodiversity can offer as a resource to improve human health and quality of life. Innovative products, to substitute classical lifelong anti-gut drugs (e.g., allopurinol or, more recently, flebuxostat) can be developed from the jungle biodiversity.

The clinical possibilities of the extract from this plant for anti-inflammatory pharmacology seem promising, although a precise chemical profile characterization is still needed. This opens several lines of phytochemical investigation that can ratify the interest for its conservation. In this case, the Nagoya Protocol cannot be forgotten and the Pakayaku Kichwa Original Community must be taken into account.

## 3. Materials and Methods

### 3.1. Ethnobotanical Survey 

The selected community lies in a fairly isolated region where bio- and ethnodiversity studies are still lacking (Bobonaza River, Pastaza, Ecuador). It is named Pakayaku. One of us (C.X.L.-Q.) was allowed to visit due to the environmental and education programs she has been conducting since 2008 from the Biological Station Pindo Mirador in the northern Bobonaza River Basin (S 1°27′09′′–W 78°04′51′′).

Plant collection permits were granted by the Ministry of the Environment. Plant vouchers were deposited at the Herbarium José Alfredo Paredes, Universidad Central de Ecuador, Quito QAP Herbarium: Ecuador, Pastaza: Sarayaku, Pakayaku, banks of the Bobonaza River, sector of Chumbi yaku, path to chacra Sra. Ana Aranda, 402 m, 01°39′36.4′′ S, 077°36′55.4′′ W, lowland evergreen forest, 2 October 2015, *C. X. Luzuriaga-Q & H. Manya* (QAP 93168). Identification was revised by C. Cerón.

The interviews and ethnobotanical protocols were as described previously [41].

Collective written research consent was granted by Ms. Luzmila Gayas, the community president of the Assembly of Pakayaku. Prior oral individual consent was obtained from the persons taking part in our survey. Planned house visits and walking routes accompanied by Kichwa interpreters and local inhabitants of Pakayaku were made. Interviews were semi-structured and included a series of open questions aimed to encourage discussion. All interviews were recorded. Four knowledgeable elders of the Pakayaku community acted as informants and agreed to reveal their wisdom of the karacha panga. The informants answered freely about several topics, namely the Kichwa common name, the parts of the plant used, the description of use, the harvest season, storage (if any), concoction, and treatment targets. After the field wok, data were added to a Microsoft Excel spreadsheet. All recorded uses were referred to a previously published classification [14]. The data provided by the community were compared with the existing ethnobotanical literature from Ecuador [14].

### 3.2. Bibliography Review and Justification of the Activity

A bibliographic study was performed to provide scientific evidence for the medicinal uses of the plant. The accessed databases were: ISI, Scopus, Dialnet, SpaceNet, MEDLINE, PubMed, ScienceDirect, Google Patents and Scholar, and Wiley Online. The methodological Prisma 2009 Flow Diagram [42] was followed.

## Figures and Tables

**Table 1:** Biological activity and chemical components identified (bibliographic references in brackets) for *Hyptis* species from the Catalogue of Useful Plants of Ecuador.

Species	Principal Component		Activities	References
*H. atrorubens* Poit.	E-caryophyllene	sesquiterpene	Anti-inflammatory	[15]
*H. capitata* Jacq.	eugenol	monoterpene	antimicrobial	[16]
*H. eriocephala* Benth.	nepetoidine	sesquiterpene	antifungal, antifeedant	[17]
*H. mutabilis* Briq.	E-caryophyllene	sesquiterpene	Anti-inflammatory	[18]
*H. obtusata* Benth.	--	---	ns	[19]
*H. pectinata*	pectinolide	furanone	antinociceptive	[20,21]
*H. purdiei* Benth.	--	--	ns	[22]
*H. recurvata* Poit.	1,8 cineol	monoterpene	Anti-inflammatory	[23]
*H. verticillata* Jacq.	17 lignans	others	antineoplasic	[24]
